# The association between lactate dehydrogenase to serum albumin ratio and the 28-day mortality in patients with sepsis-associated acute kidney injury in intensive care: a retrospective cohort study

**DOI:** 10.1080/0886022X.2023.2212080

**Published:** 2023-05-17

**Authors:** Minghao Liang, Xiuhong Ren, Di Huang, Zhishen Ruan, Xianhai Chen, Zhanjun Qiu

**Affiliations:** a College of Traditional Chinese Medicine, Shandong University of Traditional Chinese Medicine, Jinan, China; b School of Nursing, Shandong University of Traditional Chinese Medicine, Jinan, China; c First Clinical Medical College, Shandong University of Traditional Chinese Medicine, Jinan, China; d The Affiliated Hospital of Shandong University of Traditional Chinese Medicine, Jinan, China

**Keywords:** Sepsis-associated acute kidney injury, lactate dehydrogenase to albumin ratio, prognosis, MIMIC-IV, SA-AKI

## Abstract

**Background:**

The mortality rate of patients with sepsis-associated acute kidney injury (SA-AKI) in the intensive care unit (ICU) is high, and there is a need for early identification of SA-AKI patients with poor prognoses. This study investigated the relationship between the lactate dehydrogenase to serum albumin ratio (LAR) and prognosis in patients with SA-AKI.

**Methods:**

We performed a retrospective cohort study of patients with SA-AKI who are represented in the Medical Information Mart for Intensive Care IV (MIMIC-IV). We used multivariable Cox regression analysis to determine adjusted hazard ratios (HRs) and 95% confidence intervals (CIs). Subgroup analysis, survival curves, and curve fitting were used to evaluate a connection between the LAR and prognosis in patients with SA-AKI.

**Results:**

There were a total of 6453 participants in this research. The average age of the participants was 63.9 ± 16.1 years, and the average LAR was 11.0 (7.6, 17.7)/IU/g. After controlling for variables, the HRs for 28-day mortality were 1.20 (HR: 1.20, 95% CI: 1.05–1.38, *p = 0.008*) and 1.61 (HR: 1.61, 95% CI: 1.41–1.84, *p < 0.001*) for Tertile 2 (T2, 8.59≤ LAR< 14.66) and Tertile 3 (T3, LAR ≥ 14.66), respectively, compared to Tertile 1 (T1, LAR < 8.59). The outcomes for 90-day mortality and in-hospital death rate were comparable. The Kaplan–Meier (KM) analysis revealed that the group with greater LAR had higher 28-day and 90-day death rates.

**Conclusion:**

Our study shows that LAR is associated with poor prognosis in patients with SA-AKI. Higher LAR is associated with higher 28-day, 90-day, and in-hospital mortality.

## Introduction

Sepsis, an extremely dangerous condition characterized by life-threatening organ dysfunction, is caused by a dysregulated host response to infection [1] and often results in high mortality rates for patients in the intensive care unit (ICU) [2]. As a common consequence of sepsis, sepsis-associated acute kidney injury (SA-AKI) is associated with high morbidity and a high fatality rate in critically ill patients [3].

Currently, there are no disease-modifying strategies for SA-AKI that allows us to be aggressive intervention. Most of the therapeutic approach is minimizing exposure to nephrotoxins, optimizing hemodynamics, achieving the source of the primary infection, and minimizing unnecessary fluid accumulation. Therefore, clinicians should identify patients with SA-AKI who have poor prognoses [4]. Clinicians may promote early intervention to minimize mortality by using clinical signs to predict illness severity and prognosis [5].

Lactate dehydrogenase (LDH), which converts pyruvate to lactate, is a common enzyme involved in energy metabolism in cells [6]. It has been shown that metabolic reprogramming occurs during SA-AKI. Renal tubular epithelial cells (RTECs) first produce energy *via* glycolysis rather than by oxidative phosphorylation, resulting in a substantial conversion of pyruvate to lactate [7]. LDH is involved in this process. Albumin responds to systemic inflammation and nutritional status [8], and some studies have found an association between albumin levels and poor prognosis in sepsis [9] and acute kidney injury (AKI) [10]. It has been shown that the lactate dehydrogenase to serum albumin ratio (LAR) is a prognostic factor in patients with lower respiratory tract infections [11] and in those with severe infections [12]. LAR combines two clinically readily available indicators, LDH and albumin, and should be taken seriously. Therefore, we performed a retrospective cohort study in which we explored the relationship between the LAR and the prognosis of SA-AKI patients in the Medical Information Mart for Intensive Care IV (MIMIC-IV) database who were admitted to the ICU.

## Methods

### Data sources

This retrospective observational study followed the guidelines set forth in Strengthening the Reporting of Observational Studies in Epidemiology (STROBE) [13]. MIMIC-IV (Medical Information Mart for Intensive Care–IV) is a large free public database that contains comprehensive clinical information about patients at Beth Israel Deaconess Hospital, a tertiary academic medical center in Boston, Massachusetts, United States. MIMIC-IV is the result of a collaboration between Beth Israel Deaconess Medical Center and the Massachusetts Institute of Technology. The first author of this study, Minghao Liang, was enrolled in a learning program offered by the National Institutes of Health (NIH) and was granted access to the MIMIC-IV database after passing the ‘Protecting Human Research Participants’ examination (ID: 11506836). All data in this database were deidentified to remove patient information, and all of the methods used in this study were performed according to relevant guidelines and regulations. All experimental protocols were approved by the institutional review boards of Beth Israel Deaconess Medical Center (Boston, Massachusetts, United States) and the Massachusetts Institute of Technology (Cambridge, Massachusetts, United States) (Record ID: 51261101).

### Participants

Patients with sepsis who developed AKI within 7 days of ICU admission were included in the analysis. According to the MIMIC-IV database, 23,828 adult sepsis patients were hospitalized between 2008 and 2019, and 16,966 of these developed AKI. Sepsis was defined as a 2-point increase in the sequential organ failure assessment (SOFA) score and documented or suspected infection [14–16]. AKI was determined using both serum creatinine (SCr) and urine output (UO) based on the Kidney Disease Improving Global Outcomes (KDIGO) criteria [17]. Baseline SCr was defined as the patient’s lowest SCr value seven days before ICU admission or his or her first SCr value after ICU admission if no preadmission SCr was available [18]. If a patient was admitted to the ICU many times, we analyzed only the data on the first admission [19]. Patients who were released or passed away within 48 h after ICU admission were excluded from the study. We eliminated patients with end-stage renal illness identified by the International Classification of Diseases (ICD) code and patients for whom the available clinical data were inadequate [18].

### Variables

Our study included the following variables. Demographic and admission data included age, sex, ethnicity, body weight, type of insurance, UO (Day 1 of ICU admission), AKI stage, and admission score. The latter included the SOFA score, the acute physiology score (APS) III, and scores on the Charlson comorbidity index (CCI) and the Glasgow coma scale (GCS). At the time of ICU admission, vital signs (mean arterial pressure (MAP), heart rate, respiration rate, and oxygen saturation level (SPO_2_)) were assessed. The interventions consisted of mechanical ventilation, vasopressor use, renal replacement therapy (RRT), and loop diuretics administered within the first 24 h following admission to the ICU. The laboratory tests included hemoglobin and chloride levels, white blood cell (WBC) count, SCr, sodium and glucose levels, pH, platelet count, potassium, bicarbonate, and total bilirubin. Comorbidities included septic shock, congestive heart failure, diabetes, myocardial infarction, congestive heart failure, hypertension, chronic lung disease, malignant cancer, and liver disease. Infection sites included the lung, the urinary tract, the catheter, the soft tissue of the skin, and the abdominal cavity. The LAR was computed as the ratio of initial LDH (IU/L) to serum albumin (g/L) based on laboratory values upon ICU admission. If several results for the above parameters were returned within 24 h of the patient’s admission to the ICU, the first dataset was used.

### Study endpoints

The primary endpoint in this study was 28-day mortality. Secondary outcomes were death at 90 days and in-hospital mortality.

### Statistical analysis

All variables with normal or skewed distributions are presented as mean ± standard deviation (SD) or median (interquartile range [IQR]) for continuous variables and as percentages for categorical variables. In the analysis of baseline characteristics, statistically significant differences among LAR tertiles (LAR <8.59; 8.59 ≤ LAR < 14.66; LAR≥ 14.66) were tested by one-way ANOVA (for normally distributed values), the Kruskal-Wallis H test (for data with a skewed distribution) and the Chi-square test (for categorical variables). We used univariate Cox proportional hazards models to test for links between prognostic factors and 28-day mortality. Multivariate Cox proportional hazards models were developed and used to evaluate the relationship between the LAR and 28-day and 90-day mortality. The relationship between the LAR and in-hospital mortality was also evaluated using logistic regression. We constructed and used three models to adjust for confounding factors. Confounding factors in the multivariate regression model were selected based on the results of univariate regression and clinical experience. Model 1 was adjusted only for sex and age; Model 2 was further adjusted for hemoglobin, MAP, SpO_2_, platelet count, pH, WBC count, SCr, and glucose, chloride, sodium, potassium, and bicarbonate levels, and Model 3 was further adjusted for septic shock, hypertension, myocardial infarction, liver disease, AKI stage, SOFA score, APSIII, CCI, diabetes mellitus, vasopressor use, malignant cancer, congestive heart failure, use of loop diuretics, use of RRT and site of infection. We used a Cox proportional hazards regression model with a cubic spline function and a smoothed curve fit to determine the nonlinear relationship between the LAR and 28-day mortality. If the results were calculated to be nonlinear, we used a recursive algorithm to calculate the inflexion point and then constructed a two-piecewise Cox proportional hazards regression model that included the values on both sides of the inflexion point. Kaplan–Meier (KM) curves were used to compare the survival probabilities of groups with different LAR levels. Stratified and interaction analyses were conducted according to sex, age, APS III, RRT use, vasopressor use, loop diuretic use, myocardial infarction, congestive heart failure, malignant cancer, septic shock, liver disease, and AKI stage. Fewer than five percent of the covariates had missing data in any of the analyses. Because the proportion of missing data was low, no imputation was conducted. We used PASS 15 software to calculate the sample size and set the two-sided alpha = 0.05 and the expected power to 0.9. The total sample size for the three groups was calculated to be 2481, and the obtained sample size of 6453 met the statistical requirements. All analyses were conducted using R software and Free Statistics software version 1.7. P values <0.05 were deemed significant.

## Results

### Population and baseline characteristics

**Figure 1.**
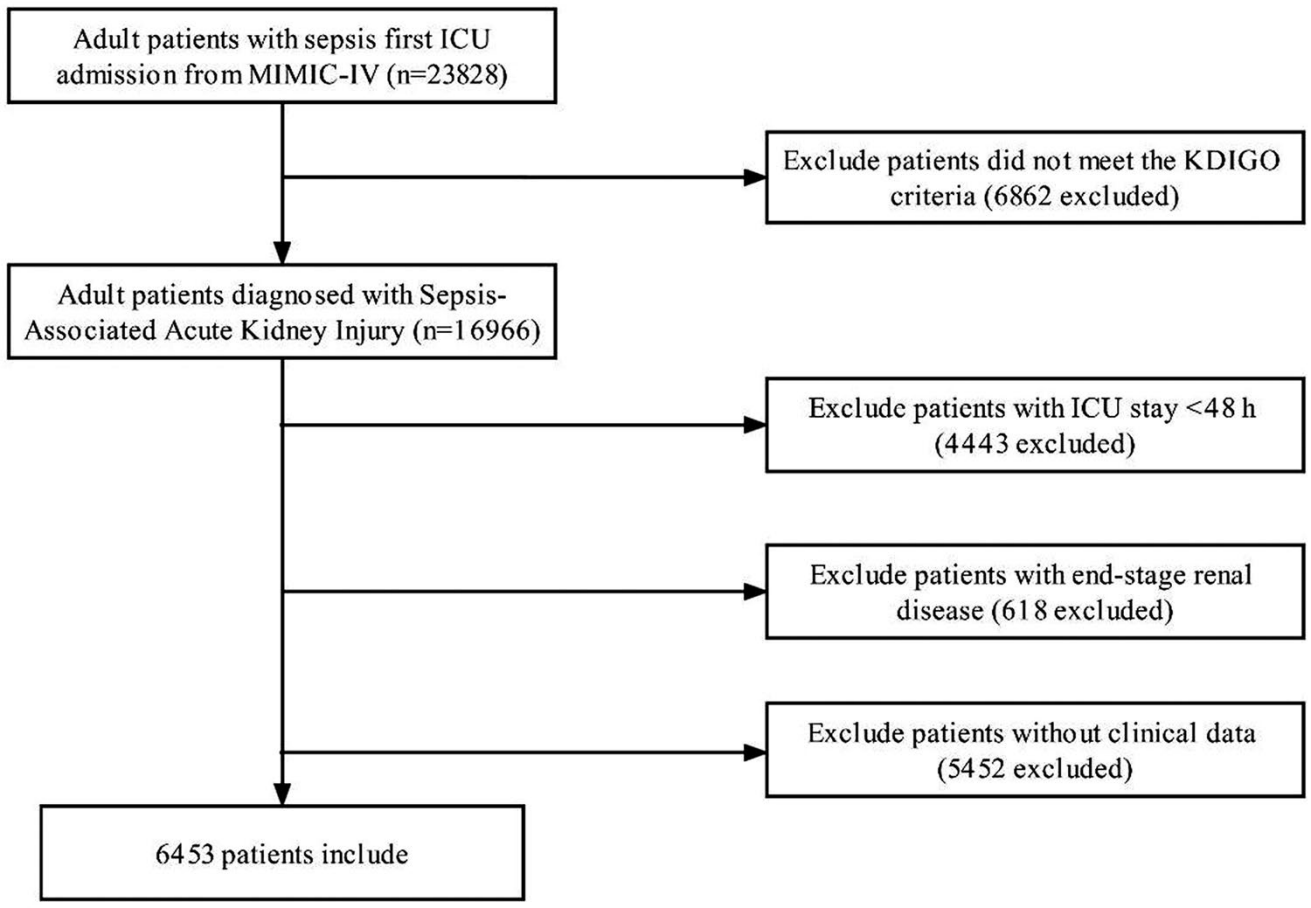
Flowchart of study patients.

Of the adult patients in the MIMIC-IV database, 23,828 satisfied the sepsis-3.0 criterion upon first ICU admission. A total of 6453 patients were ultimately included in this study (Figure 1). Table 1 summarizes the baseline characteristics of the population sorted by LAR tertiles. The mean age of the patients was 63.9 ± 16.1 years, and 58.0% were male. The 28-day, 90-day, and in-hospital death rates were 26%, 35%, and 23.9%, respectively. The patients in the highest LAR group (T3) had high values for weight, heart rate, respiration rate, SOFA score, APS III, AKI stage, WBC, LDH, LAR, SCr, glucose, and potassium. They were more likely to have ventilator use, vasopressor use, RRT use, loop diuretic use, septic shock, myocardial infarction, malignant cancer, and liver disease than were the other two groups. Participants with increased LAR had greater 28-day, 90-day, and in-hospital mortality (all *p < 0.001*) than those with lower LAR levels.

**Table 1.** Baseline characteristics of participants and outcome parameters.

Variable	Lactate dehydrogenase to albumin ratio				
		T1	T2	T3	
	Total	LAR <8.59	8.59 ≤ LAR <14.66	LAR ≥14.66	
	(*n* = 6453)	(*n* = 2150)	(*n* = 2151)	(*n* = 2152)	*p* Value
Age (years)	63.9 ± 16.1	65.4 ± 15.6	65.2 ± 15.8	61.3 ± 16.6	<0.001
Male (%)	3745 (58.0)	1273 (59.2)	1233 (57.3)	1239 (57.6)	0.396
Ethnicity, white (%)	4100 (63.5)	1441 (67)	1376 (64)	1283 (59.6)	<0.001
Weight (kg)	85.1 ± 26.7	84.9 ± 28.9	84.7 ± 25.4	85.8 ± 25.7	0.366
Medicare	2908 (45.1)	1029 (47.9)	1002 (46.6)	877 (40.8)	<0.001
UO (ml)	1310.0 (775.0, 2100.0)	1420.0 (857.0, 2175.0)	1348.0 (820.0, 2094.0)	1175.0 (630.0, 2020.0)	<0.001
AKI stage (%)					<0.001
1	1058 (16.4)	397 (18.5)	365 (17)	296 (13.8)	
2	2895 (44.9)	1086 (50.5)	1037 (48.2)	772 (35.9)	
3	2500 (38.7)	667 (31)	749 (34.8)	1084 (50.4)	
Vital signs					
Heart rate (bpm)	89.8 ± 17.6	86.1 ± 16.7	89.9 ± 17.5	93.6 ± 17.9	<0.001
MAP (mmHg)	78.8 ± 12.5	79.4 ± 11.8	78.2 ± 13.0	78.8 ± 12.5	0.007
Respiration rate (bpm)	20.5 ± 4.3	19.6 ± 3.8	20.3 ± 4.1	21.7 ± 4.6	<0.001
SPO_2_ (%)	96.9 ± 2.3	97.1 ± 2.0	97.0 ± 2.2	96.6 ± 2.7	<0.001
Scoring system, points					
SOFA score	3.0 (2.0, 5.0)	3.0 (2.0, 5.0)	3.0 (2.0, 5.0)	4.0 (3.0, 6.0)	<0.001
APS III	69.2 ± 27.6	60.6 ± 24.1	68.3 ± 26.5	78.9 ± 28.9	<0.001
CCI	6.1 ± 3.0	6.1 ± 2.9	6.2 ± 2.9	6.0 ± 3.1	0.055
GCS	14.4 ± 1.9	14.4 ± 1.8	14.3 ± 2.0	14.4 ± 1.9	0.344
Laboratory results					
Hemoglobin (g/dL)	9.7 ± 2.3	9.9 ± 2.2	9.7 ± 2.3	9.6 ± 2.3	<0.001
Platelets (k/uL)	152.0 (97.0, 220.0)	162.0 (109.0, 229.0)	158.0 (102.0, 227.0)	135.0 (78.0, 204.0)	<0.001
WBC (k/uL)	14.5 (10.1, 20.1)	13.0 (9.5, 17.8)	14.8 (10.4, 20.2)	16.1 (10.8, 22.3)	<0.001
Total bilirubin (mg/dL)	1.1 (0.6, 3.1)	0.8 (0.5, 2.1)	1.0 (0.6, 2.7)	1.7 (0.8, 4.5)	<0.001
LDH (IU/L)	309.0 (229.0, 478.0)	209.0 (176.0, 243.0)	311.0 (266.0, 366.0)	618.5 (468.0, 1060.0)	<0.001
Albumin (g/L)	29.2 ± 6.4	32.3 ± 5.9	28.6 ± 5.7	26.7 ± 6.1	<0.001
LAR (IU/g)	11.0 (7.6, 17.7)	6.8 (5.7, 7.6)	11.0 (9.7, 12.5)	23.2 (17.7, 38.9)	<0.001
SCr (mg/dL)	1.1 (0.8, 1.8)	1.0 (0.7, 1.6)	1.1 (0.8, 1.7)	1.3 (0.9, 2.1)	<0.001
Glucose (mg/dL)	134.0 (108.0, 176.0)	130.0 (106.0, 165.0)	135.0 (109.0, 177.0)	140.0 (109.0, 190.0)	<0.001
pH	7.3 ± 0.1	7.4 ± 0.1	7.4 ± 0.1	7.3 ± 0.1	<0.001
Chloride (mEq/L)	104.1 ± 7.4	103.7 ± 7.1	104.6 ± 7.4	104.0 ± 7.7	<0.001
Sodium (mEq/L)	138.3 ± 6.2	138.1 ± 6.0	138.4 ± 6.2	138.2 ± 6.4	0.184
Potassium (mEq/L)	4.3 ± 0.8	4.2 ± 0.7	4.2 ± 0.8	4.4 ± 0.9	<0.001
Bicarbonate (mEq/L)	21.6 ± 5.2	22.8 ± 5.2	21.8 ± 4.8	20.2 ± 5.1	<0.001
Interventions					
Ventilator use (%)	5128 (79.5)	1710 (79.5)	1706 (79.3)	1712 (79.6)	0.976
Vasopressor use (%)	790 (12.2)	156 (7.3)	249 (11.6)	385 (17.9)	<0.001
RRT use (%)	238 (3.7)	35 (1.6)	57 (2.6)	146 (6.8)	<0.001
Loop diuretics use (%)	691 (10.7)	175 (8.1)	230 (10.7)	286 (13.3)	<0.001
Comorbidities					
Septic shock (%)	832 (12.9)	223 (10.4)	280 (13)	329 (15.3)	<0.001
Hypertension (%)	1698 (26.3)	631 (29.3)	562 (26.1)	505 (23.5)	<0.001
Diabetes mellitus (%)	1931 (29.9)	676 (31.4)	671 (31.2)	584 (27.1)	0.002
Myocardial infarct (%)	1256 (19.5)	310 (14.4)	405 (18.8)	541 (25.1)	<0.001
Congestive heart failure (%)	2240 (34.7)	698 (32.5)	798 (37.1)	744 (34.6)	0.006
Chronic pulmonary disease (%)	1765 (27.4)	612 (28.5)	586 (27.2)	567 (26.3)	0.294
Malignant cancer (%)	997 (15.5)	294 (13.7)	325 (15.1)	378 (17.6)	0.002
Liver disease (%)	1598 (24.8)	439 (20.4)	497 (23.1)	662 (30.8)	<0.001
Site of infection					
Lung (%)	2725 (42.2)	847 (39.4)	923 (42.9)	955 (44.4)	0.003
Urinary (%)	1385 (21.5)	477 (22.2)	477 (22.2)	431 (20)	0.139
Catheter (%)	202 (3.1)	65 (3.0)	64 (3.0)	73 (3.4)	0.691
Skin soft tissue (%)	45 (0.7)	14 (0.7)	13 (0.6)	18 (0.8)	0.626
Abdominal cavity (%)	407 (6.3)	162 (7.5)	125 (5.8)	120 (5.6)	0.016
28-day mortality (%)	1728 (26.8)	388 (18)	540 (25.1)	800 (37.2)	<0.001
90-day mortality (%)	2285 (35.4)	576 (26.8)	738 (34.3)	971 (45.1)	<0.001
In-hospital mortality (%)	1542 (23.9)	327 (15.2)	467 (21.7)	748 (34.8)	<0.001

Data are presented as the mean ± SD or median (IQR) for skewed variables or numbers (proportions) for categorical variables; T1, LAR < 8.59; T2, 8.59 ≤ LAR <14.66; T3, LAR ≥14.66. bpm: beats per minute; MAP: mean arterial pressure; SOFA score: sequential organ failure assessment score; APS III: acute physiology score III; CCI: Charlson comorbidity index; GCS: Glasgow Coma Scale; WBC: white blood cell count; LDH: lactate dehydrogenase; LAR: lactate dehydrogenase to albumin ratio; SCr: serum creatinine; RRT: renal replacement treatment; AKI: acute kidney injury.

### Multivariable Cox regression analysis

After performing univariate Cox regression analysis (Table S1), we developed and used three multivariable models to identify significant correlations between the LAR and various clinical outcomes. Table 2 displays the hazard ratios (HRs) and 95% confidence intervals (CIs) for the models. In the unadjusted model for factors, the HRs for 28-day mortality for the patients in the T2 and T3 groups were 1.44 (HR: 1.44, 95% CI: 1.26–1.64, *p < 0.001*) and 2.37 (HR: 2.37, 95% CI: 2.10–2.68, *p < 0.001*), respectively, compared to the T1 reference group. After controlling for ethnicity, sex, MAP, SpO_2_, UO, age, hemoglobin, heart rate, platelet count, WBC, SCr, glucose, pH, chloride, sodium, potassium, bicarbonate, AKI stage, SOFA score, APSIII, CCI, RRT use, loop diuretics use, septic shock, diabetes mellitus, myocardial infarction, congestive heart failure, malignant cancer, hypertension, liver disease, vasopressor use and site of infection, the adjusted HRs for the T2 and T3 groups compared to T1 were 1.20 (HR: 1.20, 95% CI: 1.05–1.38, *p = 0.008*) and 1.61 (HR: 1.61, 95% CI: 1.41–1.84, *p < 0.001*), respectively. When examined as a continuous variable, the LAR (log_2_) was linked to 28-day mortality. The impact size of LAR (log_2_) on 28-day mortality increased by 46% for every unit in the unadjusted model. The effect size increased by 14% for LAR (log_2_) in the fully adjusted model (Model 3). Investigation of in-hospital mortality and mortality after 90 days yielded comparable findings.

**Table 2.** Hazard ratio (HR) [95% confidence intervals (CIs)] for mortality across groups divided according to the ratio of lactate dehydrogenase to albumin (LAR) level.

Variable	Unadjusted		Model 1		Model 2		Model 3	
	HR (95% CI)	*p* Value	HR (95% CI)	*p* Value	HR (95% CI)	*p* Value	HR (95% CI)	*p* Value
Primary outcomes								
28-day mortality								
LAR (log_2_)	1.46 (1.39−1.53)	<0.001	1.55 (1.48–1.63)	<0.001	1.37 (1.3–1.44)	<0.001	1.14 (1.09–1.18)	<0.001
T1 (LAR < 8.59)	1 (Ref)		1 (Ref)		1 (Ref)		1 (Ref)	
T2 (8.59 ≤ LAR <14.66)	1.44 (1.26−1.64)	<0.001	1.45 (1.28–1.66)	<0.001	1.40 (1.22–1.60)	<0.001	1.20 (1.05–1.38)	<0.001
T3 (LAR ≥ 14.66)	2.37 (2.1−2.68)	<0.001	2.59 (2.29–2.93)	<0.001	2.17 (1.9–2.47)	<0.001	1.61 (1.41–1.84)	<0.001
P for trend		<0.001		<0.001		<0.001		<0.001
Secondary outcomes								
90-day mortality								
LAR (log_2_)	1.37 (1.31−1.43)	<0.001	1.47 (1.41–1.54)	<0.001	1.32 (1.25–1.38)	<0.001	1.11 (1.07–1.15)	<0.001
T1 (LAR < 8.59)	1 (Ref)		1 (Ref)		1 (Ref)		1 (Ref)	
T2 (8.59 ≤ LAR <14.66)	1.35 (1.21−1.5)	<0.001	1.36 (1.22–1.52)	<0.001	1.29 (1.16–1.45)	<0.001	1.12 (1–1.26)	0.046
T3 (LAR ≥ 14.66)	2.01 (1.81−2.23)	<0.001	2.23 (2.01–2.47)	<0.001	1.91 (1.71–2.14)	<0.001	1.46 (1.3–1.63)	<0.001
P for trend		<0.001		<0.001		<0.001		<0.001
In-hospital mortality^a^								
LAR (log_2_)	1.69 (1.58−1.81)	<0.001	1.79 (1.67–1.92)	<0.001	1.56 (1.44–1.69)	<0.001	1.21 (1.14–1.28)	<0.001
T1 (LAR < 8.59)	1 (Ref)		1 (Ref)		1 (Ref)		1 (Ref)	
T2 (8.59 ≤ LAR <14.66)	1.55 (1.32−1.81)	<0.001	1.56 (1.33–1.82)	<0.001	1.46 (1.24–1.73)	<0.001	1.25 (1.05–1.49)	0.013
T3 (LAR ≥ 14.66)	2.97 (2.56−3.44)	<0.001	3.21 (2.77−3.73)	<0.001	2.56 (2.17–3.00)	<0.001	1.87 (1.57–2.23)	<0.001
P for trend		<0.001		<0.001		<0.001		<0.001

Model 1 is adjusted for sex and age.

Model 2 is adjusted as is Model 1 and is also adjusted for ethnicity, heart rate, MAP, SpO_2_, UO, hemoglobin, platelets, WBC, SCr, glucose, pH, chloride, sodium, potassium, and bicarbonate.

Model 3 is adjusted as is Model 2 and is also adjusted for AKI stage, SOFA score, APSIII, CCI, septic shock, hypertension, diabetes mellitus, myocardial infarct, congestive heart failure, malignant cancer, liver disease, loop diuretic use, RRT use, vasopressor use and site of infection.

^a^Logistic regression was used to evaluate the association between the LAR and in-hospital mortality. The results are expressed as odds ratios (95% CIs).

LAR; lactate dehydrogenase to albumin ratio; HR: hazard ratio; CI: confidence interval.

### Kaplan–Meier curves

**Figure 2.**
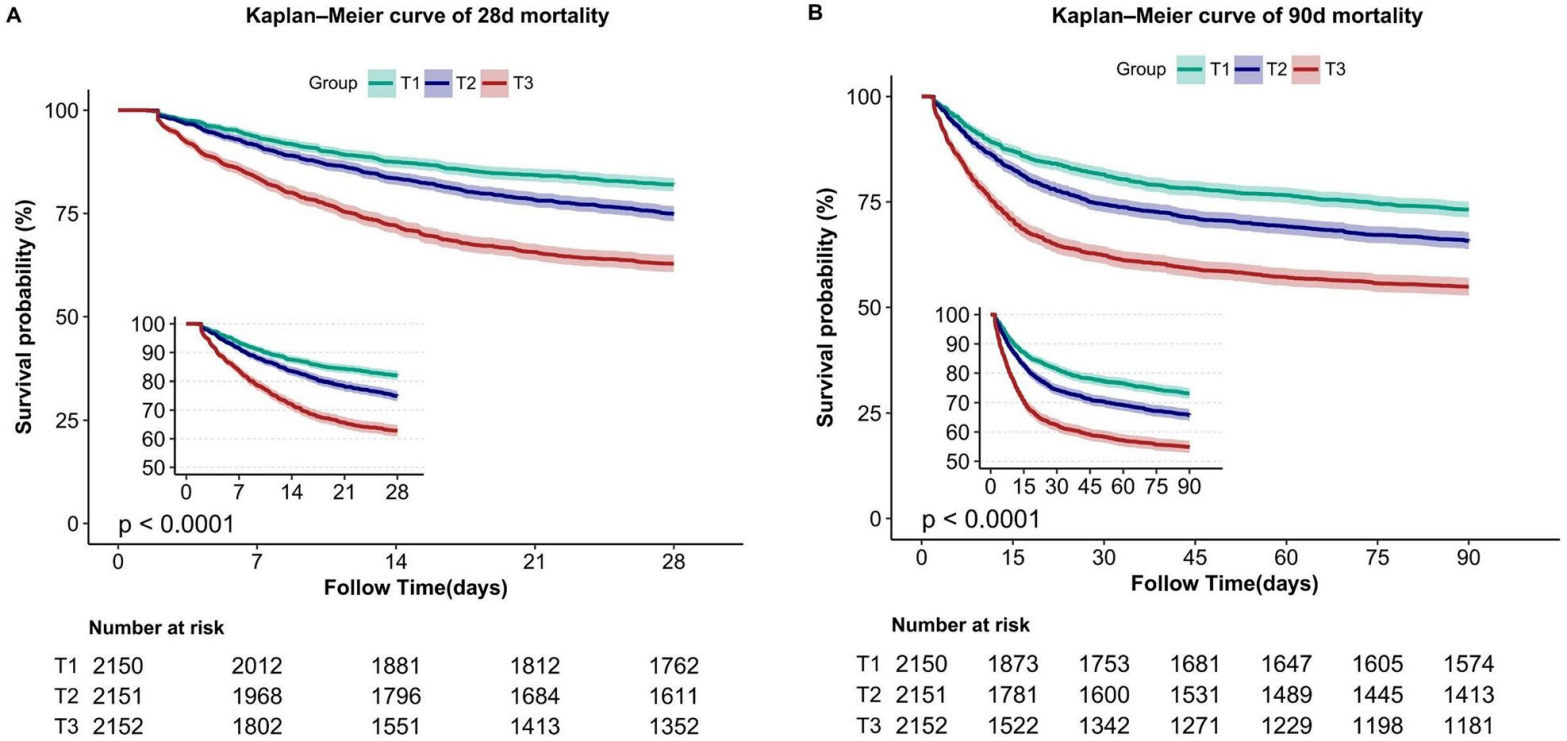
(A): Kaplan–Meier curve of 28-day mortality for patients with SA-AKI. (B) Kaplan–Meier curve of 90-day mortality for patients with SA-AKI.

To determine cumulative survival for the different LAR groups, 28-day survival curves were created for individuals with SA-AKI by categorizing them based on the LAR tertiles. The KM survival curve revealed that individuals with high LAR had substantially poorer 28-day survival and that 28-day survival decreased with decreasing baseline LAR (Figure 2). A similar finding was obtained using the 90-day mortality curve.

### Subgroup analysis and sensitivity analysis

The subgroup analysis demonstrated a correlation between the LAR and 28-day mortality for patients of various statuses, as shown in Table 3. Although myocardial infarction and liver disease showed an interaction (P for interaction <0.05), there was a positive association between the LAR and 28-day mortality in each group (HR > 1 in each group). Other subgroups exhibited no significant interactions (P for interaction > 0.05).

**Table 3.** Subgroup analysis of the association between 28-day mortality and LAR.

	No. of patients	Lactate dehydrogenase to albumin ratio			*p* for interaction
		T1	T2	T3	
		LAR < 8.59	8.59 ≤ LAR <14.66	LAR ≥ 14.66	
Sex					0.381
Male	3745	1 (Ref)	1.45 (1.21–1.74)	2.21 (1.85–2.64)	
Female	2708	1 (Ref)	1.23 (1–1.52)	1.76 (1.44–2.15)	
Age					0.078
<60	3118	1 (Ref)	1.30 (1–1.68)	1.55 (1.21–1.98)	
≥60	3335	1 (Ref)	1.37 (1.17–1.61)	2.24 (1.91–2.62)	
APS III					0.232
<42	1001	1 (Ref)	1.64 (0.93–2.88)	2.63 (1.47–4.69)	
≥42	5452	1 (Ref)	1.3 (1.13–1.49)	1.87 (1.64–2.15)	
RRT					0.299
Yes	238	1 (Ref)	1.12 (0.46–2.7)	1.23 (0.58–2.59)	
No	6215	1 (Ref)	1.36 (1.19–1.56)	2.02 (1.76–2.31)	
Vasopressor use					0.465
Yes	790	1 (Ref)	1.19 (0.79–1.78)	2.00 (1.37–2.92)	
No	5663	1 (Ref)	1.38 (1.2–1.6)	1.98 (1.72–2.28)	
Loop diuretics use					0.099
Yes	691	1 (Ref)	2 (1.33–3)	2.11 (1.41–3.16)	
No	5762	1 (Ref)	1.3 (1.12–1.5)	2 (1.74–2.3)	
Myocardial infarct					0.017
Yes	1256	1 (Ref)	1.66 (1.18–2.35)	3.13 (2.26–4.33)	
No	5197	1 (Ref)	1.31 (1.13–1.52)	1.82 (1.57–2.11)	
Congestive heart failure					0.686
Yes	2240	1 (Ref)	1.36 (1.09–1.69)	2.18 (1.75–2.7)	
No	4213	1 (Ref)	1.42 (1.19–1.68)	2.01 (1.7–2.38)	
Malignant cancer					0.436
Yes	997	1 (Ref)	1.49 (1.08–2.04)	2.67 (1.96–3.63)	
No	5456	1 (Ref)	1.32 (1.13–1.53)	1.87 (1.61–2.16)	
Septic shock					0.439
Yes	832	1 (Ref)	1.31 (0.95–1.81)	1.83 (1.35–2.49)	
No	5621	1 (Ref)	1.37 (1.18–1.59)	2.03 (1.75–2.34)	
AKI stage					0.090
1	1058	1 (Ref)	1.78 (1.19–2.66)	2.08 (1.36–3.18)	
2	2895	1 (Ref)	1.31 (1.04–1.65)	2.25 (1.79–2.83)	
3	2500	1 (Ref)	1.32 (1.09–1.59)	1.71 (1.44–2.04)	
Liver disease					
Yes	1598	1 (Ref)	1.33 (1.04–1.69)	1.47 (1.16–1.86)	<0.001
No	4855	1 (Ref)	1.37 (1.16–1.61)	2.37 (2.02–2.78)	

HRs (95% CIs) were derived from Cox proportional hazards regression models. Each stratification was adjusted for all factors included in Model 3 in Table 2 except the stratification factor itself. APS III, acute physiology score; RRT, renal replacement treatment; AKI, acute kidney injury.

### Analyses of the nonlinear relationship

**Figure 3.**
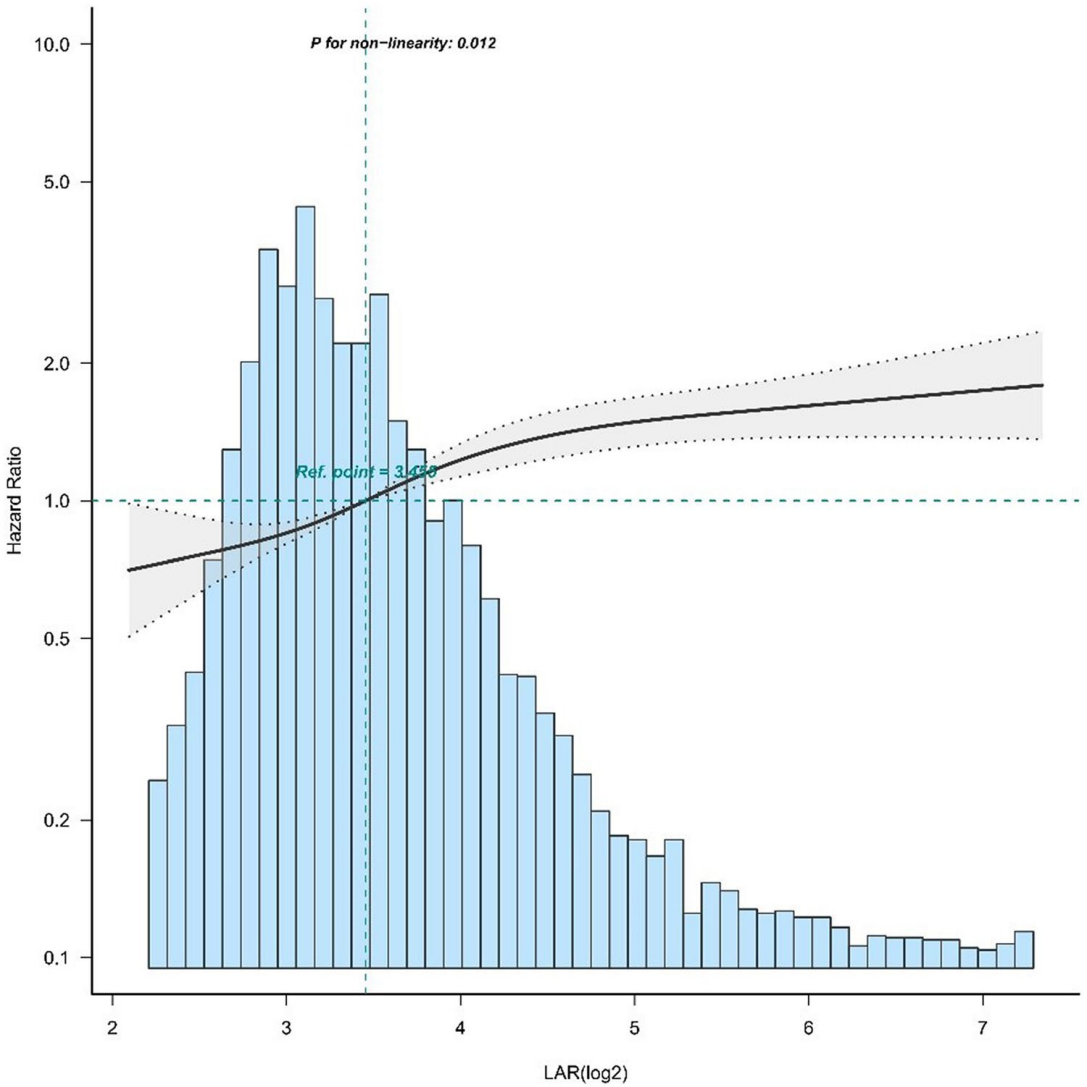
Curve fitting of the LAR (log_2_) and 28-day mortality in patients with SA-AKI.

After correction for confounding variables, restricted cubic spline analysis revealed that the association between the LAR (log_2_) and 28-day mortality was nonlinear (Figure 3). We used the two-piecewise Cox proportional hazard model to fit the link between the LAR (log_2_) and 28-day mortality since the P value for the log-likelihood ratio test was less than 0.05. Using the recursive method with the two-part Cox proportional hazard model, the inflection point was determined to be 5.57. Within the inflection point of 5.57, a more significant positive connection between the LAR and 28-day mortality was observed; 28-day mortality increased by 29% for each unit LAR (log_2_) increase. When the LAR exceeded 5.57, there was no significant difference in 28-day mortality (Table 4).

**Table 4.** Threshold effect analysis of the relationship between the LAR (log_2_) and 28-day mortality in patients with SA-AKI.

Threshold of LAR (log_2_)	HR (95% CI)	*p* Value
<5.57	1.29 (1.20, 1.38)	<0.001
≥5.57	1.00 (0.84, 1.20)	0.95
Likelihood ratio test		<0.001

The data have been adjusted for all of the factors included in Model 3 in Table 2.

## Discussion

The results of this study show that the LAR is an independent predictor of 28-day, 90-day, and in-hospital death in ICU patients with SA-AKI. LAR (log_2_) and 28-day mortality of SA-AKI patients in intensive care exhibited a nonlinear association. In patients with SA-AKI, survival decreases with increasing LAR.

The enzyme LDH is involved in energy metabolism and converts pyruvate to lactate in cells. It has been shown that LDH is an independent predictor of prognosis in individuals with sepsis [20] and AKI [21]. Inflammation, metabolic recoding, RTEC apoptosis, and shock play essential roles in SA-AKI [7]. RTECs, the most metabolically active cells in the kidney, undergo metabolic recoding in SA-AKI. Although aerobic respiration is normally the main mechanism of cellular energy production, in SA-AKI, RTECs first undergo glycolysis to convert pyruvate to lactate [22]. LDH activity increases during this process. During SA-AKI, infiltration by inflammatory cells and the presence of large amounts of inflammatory factors lead to the deterioration of renal function [23], and apoptosis of RTECs leads to the release of lactate dehydrogenase, resulting in elevated lactate dehydrogenase levels.

Albumin is an indicator of inflammation and systemic nutritional status, and studies have shown that albumin is associated with the prognosis of patients with sepsis [24]. Hypoproteinemia is considered an independent risk factor for the prognosis of AKI [25]. Some evidence suggests that albumin has a renoprotective mechanism. First, albumin may clear reactive oxygen species and prevent oxidative damage [26]. Second, albumin is essential in maintaining renal perfusion and glomerular filtration [27]. Finally, further studies have demonstrated that albumin can stimulate DNA synthesis in renal tubular cells through a signalling pathway involving Ca^2+^ [28]. Albumin plays a vital role in protecting the kidney and maintaining renal function. Lower albumin levels often indicate poor prognosis in individuals with AKI.

It has been shown that LAR is an independent risk factor for poor prognosis in patients with lower respiratory tract infections [11]. Further studies have indicated that LAR is associated with prognosis in patients with COVID-19 [29] and in those with nasopharyngeal carcinoma [30]. No studies that address whether the LAR can be used to assess the prognosis of patients with SA-AKI have been published. LAR uses two indicators, LDH and albumin, to determine the prognosis of patients with SA-AKI in terms of metabolic recoding, degree of inflammation, and nutritional status. Our study showed that LAR was associated with poor prognosis in patients with SA-AKI. This relationship remained stable when multivariate and subgroup analyses were used to reduce the effects of confounding factors.

Some limitations of this study must be considered. First, this is a retrospective study with some inevitable bias, and we adjusted some variables to ensure that the results were as accurate as possible. Second, we included only the values of the first LAR after ICU admission and did not follow its dynamics; however, the first LAR may more accurately predict the prognosis of SA-AKI. Finally, we included only patients who were admitted to the ICU for the first time, and this may have led to selection bias. Therefore, prospective studies are needed to validate our conclusions. Despite these drawbacks, our study of how the LAR is related to prognosis in individuals with SA-AKI is of interest.

## Conclusion

LAR is a possible prognostic factor in patients with SA-AKI and is associated with poor outcomes. The greater the LAR is, the greater the 28-day, 90-day, and in-hospital death rates.

## Supplementary Material

Supplemental Material

## Data Availability

In this work, we investigated publicly accessible datasets. These datasets are available at https://physionet.org/content/mimiciv.
