# Supplementary material for: The association between lactate dehydrogenase to serum albumin ratio and the 28-day mortality in patients with sepsis-associated acute kidney injury in intensive care: a retrospective cohort study
Source: Ren Fail. 2023 May 17;45(1):2212080. doi: 10.1080/0886022X.2023.2212080 (PMC10193884; doi:10.1080/0886022X.2023.2212080)
Supplement: Supplemental Material [file IRNF_A_2212080_SM1429.pdf]

Table S1 Results of univariate analysis of 28-day mortality.

**Characteristic**	HR (95% CI)	***P value***
Age	1.02 (1.01, 1.02)	< 0.001
Gender	0.96 (0.88, 1.06)	0.455
Ethnicity	0.78 (0.71, 0.86)	< 0.001
Weight	0.9954 (0.9934, 0.9974)	< 0.001
Insurance	1.25 (1.14, 1.37)	< 0.001
UO	0.9997 (0.9996, 0. 9997)	< 0.001
**AKI stage**		
1	(Ref)	
2	1.24 (1.04, 1.47)	0.014
3	2.97 (2.52, 3.5)	< 0.001
Heart rate	1.0035 (1.0008, 1.0062)	0.01
MAP	0.9923 (0.9889, 0.9957)	< 0.001
Respiration rate	1.04 (1.03, 1.05)	< 0.001
SPO_2	0.95 (0.93, 0.96)	< 0.001
SOFA score	1.08 (1.06, 1.1)	< 0.001
APS III	1.02 (1.02, 1.02)	< 0.001
CCI	1.14 (1.12, 1.15)	< 0.001
Hemoglobin	0.95 (0.93, 0.97)	< 0.001
Platelets	0.9997 (0.9992, 1.0001)	0.144
WBC	1.0045 (1.0024, 1.0067)	< 0.001
LDH	1.0001 (1.0001, 1.0001)	< 0.001
Albumin	0.97 (0.96, 0.98)	< 0.001
LAR	1.0024 (1.0019, 1.003)	< 0.001
SCr	1.1 (1.07, 1.13)	< 0.001
Glucose	1.0097 (1.0003, 1.0193)	0.044
pH	0.2 (0.13, 0.3)	< 0.001
Chloride	0.98 (0.97, 0.99)	< 0.001
Sodium	0.9907 (0.9831, 0.9983)	0.017
Potassium	1.14 (1.08, 1.2)	< 0.001
Bicarbonate	0.97 (0.96, 0.98)	< 0.001
Ventilator use	0.91 (0.82, 1.03)	0.125
Vasopressor use	1.53 (1.35, 1.74)	< 0.001
RRT	1.48 (1.19, 1.84)	< 0.001
Loop diuretics use	1.3 (1.13, 1.5)	< 0.001
Septic shock	1.67 (1.48, 1.89)	< 0.001
Hypertension	0.85 (0.76, 0.95)	0.003
Diabetes mellitus	0.98 (0.89, 1.09)	0.741
Myocardial infarct	1.23 (1.09, 1.37)	< 0.001
Congestive heart failure	1.17 (1.06, 1.29)	0.002
Chronic pulmonary disease	1.06 (0.95, 1.18)	0.273
Malignant cancer	1.67 (1.49, 1.87)	< 0.001
Liver disease	1.56 (1.41, 1.72)	< 0.001

Information Classification: General

MAP, mean arterial pressure; SOFA score, sequential organ failure assessment score; APS III, acute physiology score III; CCI, Charlson comorbidity index; WBC, white blood count; LDH, lactate dehydrogenase; LAR, lactate dehydrogenase to albumin ratio; SCr, serum creatinine; RRT, renal replacement treatment; AKI, acute kidney injury.

Information Classification: General
